# The Effects of Hybridization on the Flexural Performances of Carbon/Glass Interlayer and Intralayer Composites

**DOI:** 10.3390/polym10050549

**Published:** 2018-05-20

**Authors:** Weili Wu, Qingtao Wang, Amos Ichenihi, Yongmao Shen, Wei Li

**Affiliations:** 1 College of Textiles, Donghua University, NO. 2999, Northern RenminRd, Songjiang District, Shanghai 201620, China; 1152003@mail.dhu.edu.cn (W.W.); a19870628wqt@126.com (Q.W.); amil.iche@gmail.com (A.I.); 2 Key Lab of Textile Science & Technology, Ministry of Education, NO. 2999, Northern RenminRd, Songjiang District, Shanghai 201620, China; 3 Center for Civil Aviation Composites, NO. 2999, Northern RenminRd, Songjiang District, Shanghai 201620, China; 4 Shanghai PUMA M&E Engineering Technology Co., Ltd., NO. 897, Zhongjiang Road, Putuo District, Shanghai 200062, China; 15026503803@163.com

**Keywords:** carbon/glass hybrid composites, flexural properties, interlayer hybrid, intralayer hybrid

## Abstract

The effects of hybridization on the flexural properties of interlayer and intralayer Carbon/Glass (C/G) composites were explored in this work. First, the theoretical analysis of stress distribution on interlayer hybrid composites was discussed. The experimental results showed that the layer structure is the critical factor affecting the flexural properties for interlayer hybrid composites, and the mixed ratio has no obvious impact. Since the carbon fiber is distributed at the top or bottom surface, the interlayer composites can obtain the maximum flexural modulus. Some structures can even achieve the same modulus as the pure carbon composites, and an excellent flexural strength can be attained with the carbon fiber located in the bottom layer. In terms of the intralayer hybrid composites, the fracture strain, flexural modulus, and flexural strength basically change linearly as the glass fiber content increases, which is consistent with the calculated values via the rule of mixture (ROM). Additionally, the C/G mixed ratio has a decisive effect on the flexural properties of intralayer hybrid composites; however, they are affected weakly by the layer structure. In general, some structures of the interlayer hybrid composites exhibit better flexural properties than that of the intralayer hybrid composites at the same C/G hybrid ratio; the alterations in layer structures make it possible to obtain excellent flexural properties for interlayer hybrid composites with less carbon fiber.

## 1. Introduction

Applications of carbon fiber composites in fan blades have attracted the interest of many developers due to the increasing demand for larger fan blades. In addition to ensuring the high stiffness of long blades, one of the necessary factors for the use of carbon fiber is to reduce the weight [1]. However, the cost of carbon fiber is high, which may be the reason why wide applications in the industry is limited [2]. Glass fiber has a lower cost than carbon fiber; its low density and superior mechanical properties are why it is used in many fields, but its fracture strength and modulus are insufficient. Carbon fiber can provide high strength and stiffness [3,4]. Thus, it is proposed that intermingling carbon fiber into glass fiber-reinforced composite can make a composite with balanced properties that attains better mechanical performances [5]. Such a composite, consisting of two or more reinforcement fibers in a material, is called hybrid composite, which provides an effective method to minimize the cost of the composite [6]. Carbon/Glass (C/G) hybrid composites incorporate the advantages of both carbon and glass fiber to achieve the goal of improving strength and modulus of composites with less carbon fiber content [7,8]. Currently, existing studies on C/G hybrid composites mainly focus on the study of short fibers, sandwich, interlayer, and intralayer hybrids, where interlayer and intralayer hybrids are the primary hybrid forms [9].

The flexural property is one of the important mechanical properties, which remains a key factor in the design and manufacture of composite-structure parts. The flexural properties of composites are primarily determined by the compression properties of the upper layer and the tensile properties of the lower layer [10]. Therefore, for the tensile and compressive properties of different materials, the bending properties of the composites can be improved via laminate optimization [11]. Jesthi found that the replacement of glass fiber by carbon fiber produced an increase by 62% in the flexural modulus compared to the pure glass fiber composite, and the flexural fracture strain was improved by 25% [12]. Prusty reported that, with 51% carbon fiber content, the strength of a hybrid composite can reach 96% of a pure carbon fiber composite [13]. In terms of the interlayer hybrid composite, the stacking sequence that fiber distributes in the top or bottom layers is the decisive parameter that affects the flexural behavior [14,15,16,17]. Sudarisman revealed that the flexural properties of hybrid composites could be improved as glass fiber distributes in the upper layer [18]. Zhang revealed that the maximum flexural strength can be attained with the carbon layers at the exterior [19]. In addition to the layup structure, the mixed ratio plays a critical role in determining the flexural properties [20]. Dong [16] found the flexural strength increases as the glass fiber content increases, which is further demonstrated by IL Kalnin [21]. Beyond experimental investigations, the finite element method was adopted to simulate the bending properties of hybrid composites. Dong developed a simple mathematical rule to obtain the flexural modulus of hybrid composites, yet the modulus obtained from FEM is above the value obtained from experiments [14]. Kalantari used the simulation method to optimize the flexural strength, and the application of these optimal stacking sequences helps for the achievement of a positive hybrid effect [22].

However, while most of above work emphasizes the interlayer hybrid structures, little work has been done on the intralayer hybrid structures except for Hansan [20], who explored the intralayer form, which is limited to a few intralayer structures. In this paper, the flexural properties of interlayer and intralayer hybrid composites with various hybrid structures and mixed ratios were designed and studied via the experimental method and compared with the values from the theory analysis.

## 2. Materials and Methods

### 2.1. Materials

**Figure 1:**
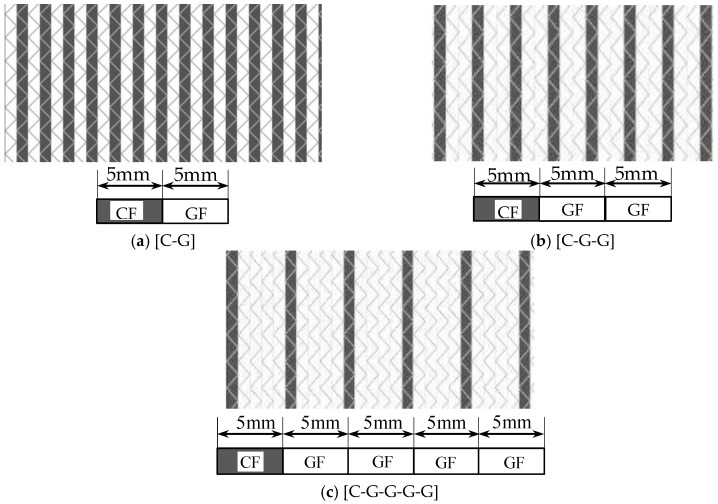
Schematic Structures of three types of non-crimp fabrics.

Five kinds of hybrid non-crimp fabrics (NCFs) of various C/G mixed ratios, including the carbon fiber fabric, the glass fiber fabric, and three C/G hybrid fabrics, were prepared and reported in Table 1. Carbon fiber was supplied by TORAY Inc., (Tokyo, Japan). glass fiber was from CPIC glass fiber Inc., (Chongqing, China) and the epoxy resin was from SWANCOR Inc. (Shanghai, China). The mechanical parameters of the raw materials are listed in Table 2, and the structures of three hybrid fabrics are illustrated in Figure 1.

### 2.2. Layer Structure Schemes of Interlayer and Intralayer Hybrid Structures

Interlayer hybrid structures were designed according to various stacking sequences of carbon fiber and glass fiber layers, as shown in Table 3. Intralayer hybrid structures are formed through the variations in the dislocation arrangements of carbon and glass fiber in various layers, which reflect the difference in dispersion degrees of the carbon and glass materials, as presented in Table 4.

### 2.3. Experiments

**Figure 2:**
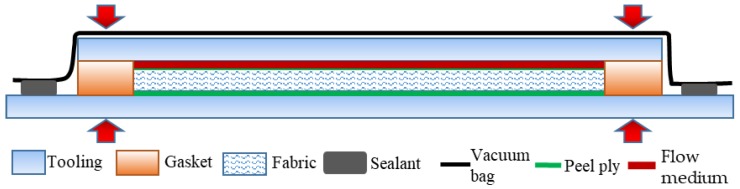
Schematic diagram of vacuum-assisted resin transfer molding (VARTM) process.

The vacuum-assisted resin transfer molding (VARTM) process was adopted to prepare composite specimens, and the fiber volume fraction was maintained at 50%. The schematic diagram of the setup is shown in Figure 2. The mold cavity was kept a height by the gaskets and sealed with a vacuum bag, and clamps were adopted to apply pressure on the molds to prevent the preform from springing up. Air was evacuated by a vacuum pump, and the resin was infused into the fabric. The curing condition was 120 °C for 8 h.

Five specimens of each laminate were tested, and three-point bending tests were performed at the speed of 1 mm/min following the standard ASTM D7264. The force attenuation rate was given for 50% as the testing end-parameter considering the difference in failure speeds and failure modes of carbon and glass fiber composites. 

**Figure 3:**
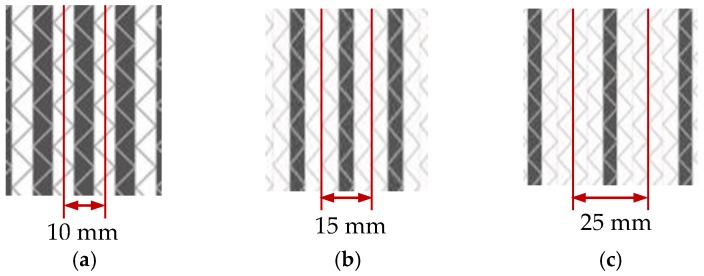
Schematics of sample cutting: (**a**) [C-G]; (**b**) [C-G-G]; (**c**) [C-G-G-G-G].

The ratio of span-to-thickness was set to 20, and the width of interlayer hybrid specimens was 13 mm. With regards to intralayer hybrid composites, the specimen width was established at the width of minimum repeating unit presented in Figure 3. In addition, carbon and glass fiber in a specimen should be symmetrically distributed.

The thickness of each layer is 0.8 mm; therefore, the dimensions of each laminate are not the same, for they depend on the number of layers and the hybrid forms. The size parameters of composites are listed in Table 5.

### 2.4. Theoretical Analysis Method

**Figure 4:**
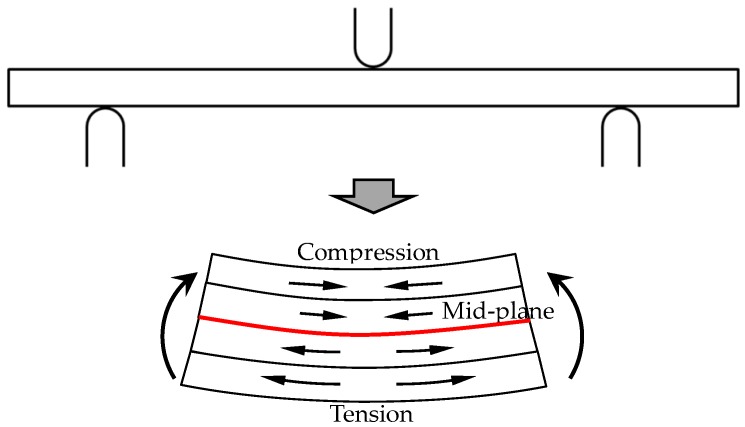
The force diagram subjected to the three-point bending.

While subjected to the three-point bending loading, deformation occurs with the stress state presented in Figure 4. The flexural property of a laminate is determined by the compression property of upper layers and the tensile property of lower layers, while the red line indicates the plane of zero stress. Since the tensile strength of composites is usually superior to the compression strength, the flexural failure is highly correlated with the compressive property of upper layers [14]. 

Calculations of flexural properties are as below:

(1) $$\sigma_{f}=\frac{3P\cdot l}{2b\cdot h^{2}};\;E_{f}=\frac{l^{3}\cdot \Delta P}{4b\cdot h^{3}\cdot \Delta S};\;\varepsilon =\frac{6S\cdot h}{l^{2}}$$

where $\sigma_{f}$ is flexural strength (MPa); *P* is failure load (N); *L* is span (mm); *h* is the thickness of the specimen (mm); *b* is the width of specimen (mm); $E_{f}$ is the flexural modulus (GPa); ΔP and ΔS is the load increment and deflection increment of the initial load-deflection curve; ε is strain; *S* is the mid-point deflection of the span (mm).

**Figure 5:**
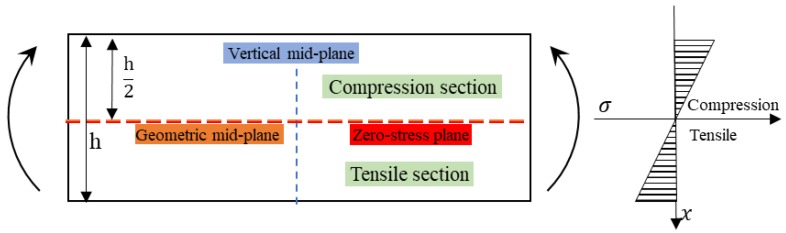
Bending stress distribution of a homogeneous composite.

According to the force equilibrium equation, as an homogenous composite is subjected to bending loading, the section above the mid-plane is subjected to compressive stress and the section below the mid-plane is subjected to the tensile stress, where the geometric mid-plane and the zero stress plane are coincident [22]. The stress distribution along the vertical mid-plane is shown in Figure 5. It can be found that, with the location along the vertical mid-plane moving from the zero-stress plane to the upper surface and the lower surface, the stress increases linearly, and the sum of the stress in the compression section and the tensile section is zero. The force equation along the vertical mid-plane is shown in the Equation (2). When the composite reaches the bending failure, the bending strength, modulus, and fracture strain can be derived from the Equation (1), since the stress on the upper and lower surface are the same, and the bending strength is the maximum stress on the upper and lower surface.

(2) $$\int_{0}^{\frac{h}{2}}E_{T}\varepsilon_{\left(x\right)}dx+\int_{0}^{-\frac{h}{2}}E_{C}\varepsilon_{\left(x\right)}dx=0$$

where *E*_T_-tensile modulus of composites; *E*_C_-compression modulus of composites; $\varepsilon_{\left(x\right)}$-strain of various locations on the vertical mid-plane.

**Figure 6:**
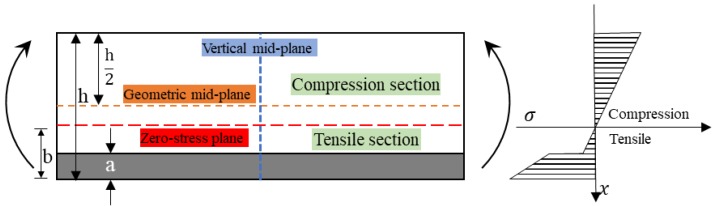
Bending stress distribution of an interlayer hybrid composite with high performance fiber distributed at the bottom surface.

However, for hybrid composites, the difference of tensile and compressive modulus of two fiber reinforcements results in the zero-stress plane deviating from the horizontal plane. Figure 6 shows the bending stress distribution of an interlayer hybrid lamina in which the bottom layer locates the high modulus fiber and the top layer is the low modulus fiber. The force equation along the vertical axis is Formula (3). From the stress distribution, it can be seen that the mixing of two reinforcements makes the zero-stress plane move downward, and more low modulus fiber layers assume the compression stress. The stress does not show a linear trend with the change of position along the vertical mid-plane, and the stress on the lower surface is greatly larger than that of the upper surface; thus, the calculation of bending strength, modulus, and fracture strain of the hybrid composite is relatively complex.

(3) $$\int_{0}^{b-a}E_{\mathrm{LT}}\varepsilon_{\left(x\right)}dx+\int_{b-a}^{b}E_{\mathrm{HT}}\varepsilon_{\left(x\right)}dx+\int_{0}^{b-h}E_{\mathrm{LC}}\varepsilon_{\left(x\right)}dx=0$$

where *E*_LT_ is the tensile modulus of low modulus composite; *E*_HT_ is the tensile modulus of high modulus composite; *E*_LC_ denotes the compression modulus of composite; ε_(*x*)_ is the strain at different locations along the vertical mid-plane.

**Figure 7:**
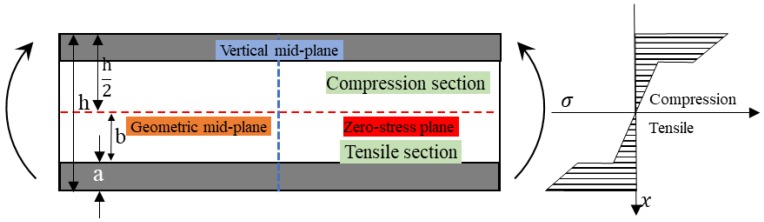
Stress distribution of a hybrid composite with high performance layers sandwiching low performance layers.

Figure 7 shows the stress distribution of the interlayer hybrid composite with a symmetric sandwich structure. The exterior deep color indicates the high modulus fiber layers, the interior is comprised of low modulus fiber layers, and the geometric mid-plane coincides with the zero-stress plane. The force equation is Equation (4). It was found that as the high modulus fiber distributes at the exterior surface and assumes the large compressive and tensile stress, the bending properties of the composites could fully exert. The maximum stress points of hybrid composites locate at the upper and lower surfaces, and their stress is the same.

(4) $$\int_{0}^{b}E_{\mathrm{LT}}\varepsilon_{\left(x\right)}dx+\int_{b}^{\frac{h}{2}}E_{\mathrm{HT}}\varepsilon_{\left(x\right)}dx+\int_{0}^{-b}E_{\mathrm{LC}}\varepsilon_{\left(x\right)}dx+\int_{-b}^{b-h}E_{\mathrm{HC}}\varepsilon_{\left(x\right)}dx=0$$

**Figure 8:**
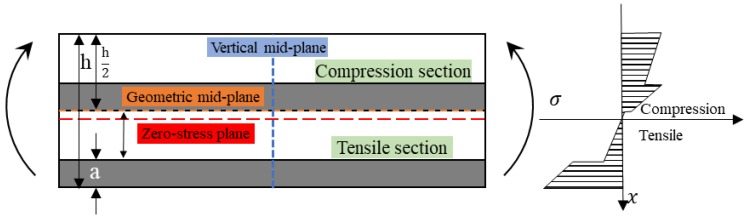
Stress distribution of a hybrid composite with low performance layers sandwiching high performance layers.

**Figure 9:**
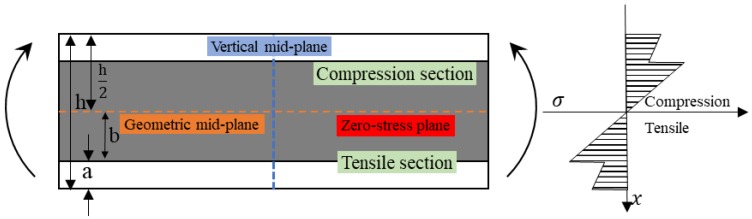
Stress distribution of an interlayer hybrid composite.

Figure 8 shows the stress distribution of the low modulus fiber sandwiching the high modulus material with the force formula, Equation (5). In this case, since the low modulus fiber assumes the maximum stress at the exterior surface, the lamina tends to fail early and the bending properties are poor. Meanwhile, the maximum stress points are not locating at the exterior surface, but at the interface of two hybrid reinforcements. For an asymmetric interlayer hybrid composite, the stress distribution is shown in Figure 9. The distribution of the maximum stress points is more complicated.

(5) $$\int_{0}^{b}E_{\mathrm{HT}}\varepsilon_{\left(x\right)}dx+\int_{b}^{\frac{h}{2}}E_{\mathrm{LT}}\varepsilon_{\left(x\right)}dx+\int_{0}^{-b}E_{\mathrm{HC}}\varepsilon_{\left(x\right)}dx+\int_{-b}^{b-h}E_{\mathrm{LC}}\varepsilon_{\left(x\right)}dx=0$$

From the above analysis, it can be concluded that the stress of hybrid composites is relatively complex. It is complicated to evaluate the bending properties of hybrid composites via the maximum stress points. In order to compare the bending properties of hybrid composites with different hybrid structures and hybrid ratios, Kalantari [22], Dong [14,17], and Jones [23] adopted the apparent flexural strength, apparent flexural modulus, and flexural fracture strain, which is Equation (1). Therefore, here the bending properties of C/G hybrid composites are still calculated according to Equation (1).

## 3. Results and Discussions

### 3.1. Flexural Properties of Interlayer Hybrid Composites

**Figure 10:**
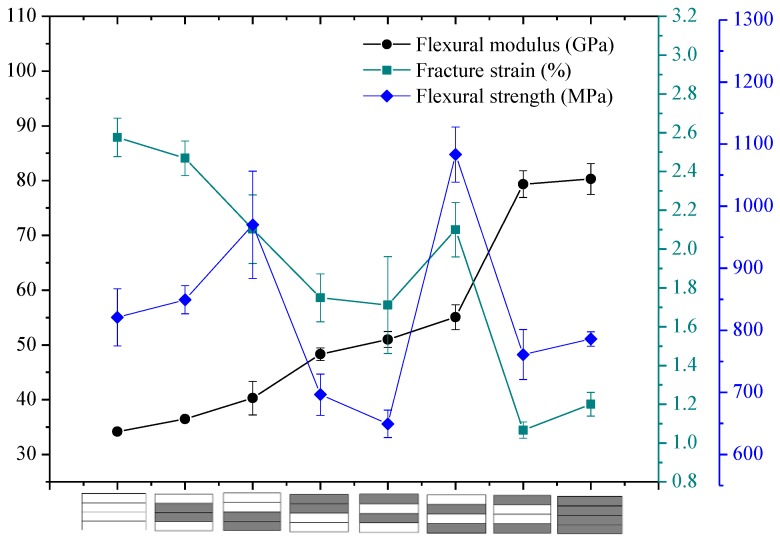
Flexural modulus, fracture strain, and strength of interlayer hybrid composite with C/G = 1:1.

**Figure 11:**
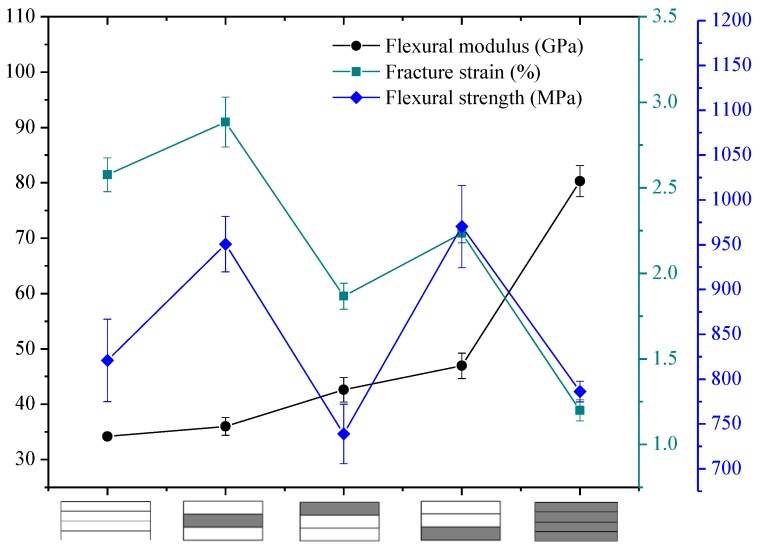
Flexural modulus, fracture strain, and strength of interlayer hybrid structure with C/G = 1:2.

**Figure 12:**
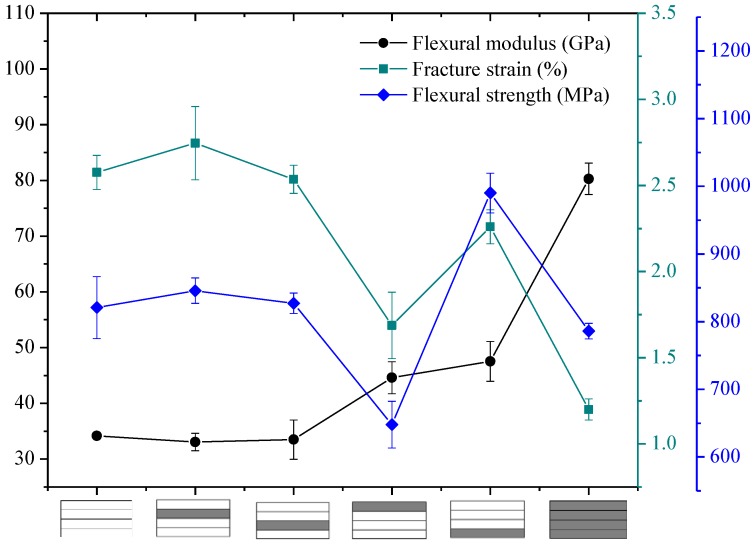
Flexural modulus, fracture strain, and strength of interlayer hybrid composite with C/G = 1:3.

**Figure 13:**
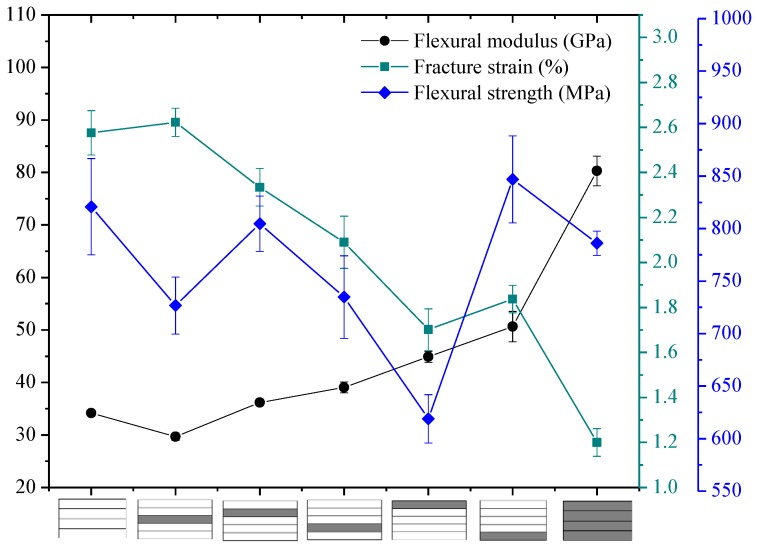
Flexural modulus, fracture strain, and strength of interlayer hybrid composite with C/G = 1:4.

The flexural properties, including the flexural strength, modulus, and fracture strain of interlayer hybrid composites with various mixed ratios and stacking sequences, are presented in Figure 10, Figure 11, Figure 12 and Figure 13.

The flexural properties of interlayer hybrid structure with C/G = 1:1 are shown in Figure 10. As revealed from the results, the flexural modulus of interlayer hybrid composites with both carbon layers distributed at the exterior surface, such as the structure [C/G/G/C], is the maximum, which basically reaches the value of the pure carbon composites, whereas the flexural failure strain is the minimum, and the strength is small. Conversely, the flexural modulus with the glass layers distributed at the top and bottom surface, such as [G/C/C/G], is lowest, and the failure strain is highest. In addition, the flexural strength with the carbon fiber distributed in the lower layers and the glass fiber in the upper layers, such as [G/G/C/C], is superior to the structure with the carbon fiber in the upper layers and glass fiber in the lower layers, such as [C/C/G/G].

While the tensile and compression modulus of carbon fiber composites surmount that of glass fiber composites, the fracture strain exhibits a different case, for the fracture strain of glass fiber is higher than that of carbon fiber. In addition, the tensile strength is above the compression strength; therefore, the compression failure of the upper layers leads to the bending failure and the mechanical decay under the bending load. The flexural fracture strain and the modulus of interlayer hybrid composites is primarily determined by the compression modulus and fracture strain of the upper layers and the tensile modulus of the lower layers, so that the flexural fracture strain of interlayer hybrid structure with the carbon fiber distributed in the upper layers is low.

Figure 11, Figure 12 and Figure 13 present the flexural properties of interlayer hybrid structures containing one carbon fiber layer, C:G = 1:2, C:G = 1:3, C:G = 1:4. Since the plane of zero stress of hybrid composites with the carbon fiber distributed in the bottom layer is slightly below the geometric mid-plane, excellent flexural modulus and strength can be attained due to more glass fiber layers assuming the compression force. In contrast, the carbon fiber located at the top layers results in the plane of zero stress moving above the geometric mid-plane and contributes to a poor flexural strength due to the premature compressive failure of the carbon layer. With the carbon fiber located in the mid-plane, a poor tensile and compression modulus of glass fiber in the exterior surface makes the flexural modulus of hybrid composites low.

**Figure 14:**
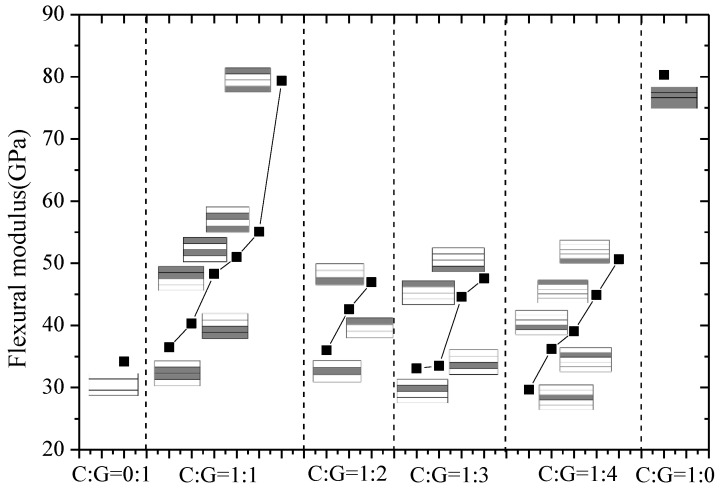
Flexural modulus of interlayer hybrid structures.

**Figure 15:**
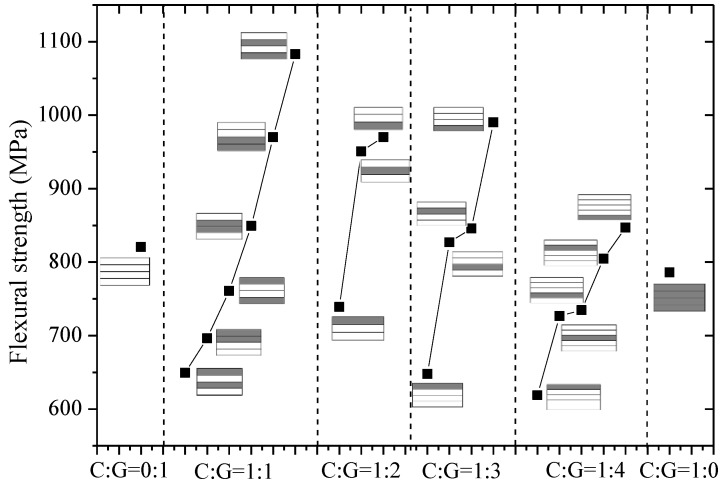
Flexural strength of interlayer hybrid structures with various stacking sequences and C/G hybrid ratios.

The flexural modulus and strength of interlayer hybrid composites with various hybrid structures and mixed ratios were compared in Figure 14 and Figure 15, separately. In Figure 14, the layer structure is the determining factor affecting the flexural modulus, while the effect of the hybrid ratio on the flexural modulus is small. For four C/G mixed ratios, with the carbon layer distributed at the top or bottom surface, the structure reaches the maxmium flexural modulus, especially for the hybrid ratio C:G = 1:1; the structure [C/G/G/C] can basically achieve the identical flexural modulus as the pure carbon composites. When carbon fiber is located in the mid-plane, the flexural modulus exhibits a minimum level. Therefore, through reasonable mixed ratio and hybrid structure optimization, the flexural modulus of hybrid composites can reach a higher level.

Figure 15 told us there is no evident impact exerted by the hybrid ratio on the flexural strength for interlayer hybrid composites; moreover, with a reasonable stacking configuration of the carbon and the glass layers, a better flexural strength than the pure carbon and glass fiber composites can be obtained. When the carbon fiber is distributed at the bottom layer, the bending strength is relatively high; when the carbon fiber is distributed at the top surface, the bending strength is minimum, which is mainly attributed to the lower compression fracture strain of carbon fiber. When carbon fiber is distributed in the middle layer, the strength is at a medium level. 

### 3.2. Flexural Properties of Intralayer Hybrid Composites

**Figure 16:**
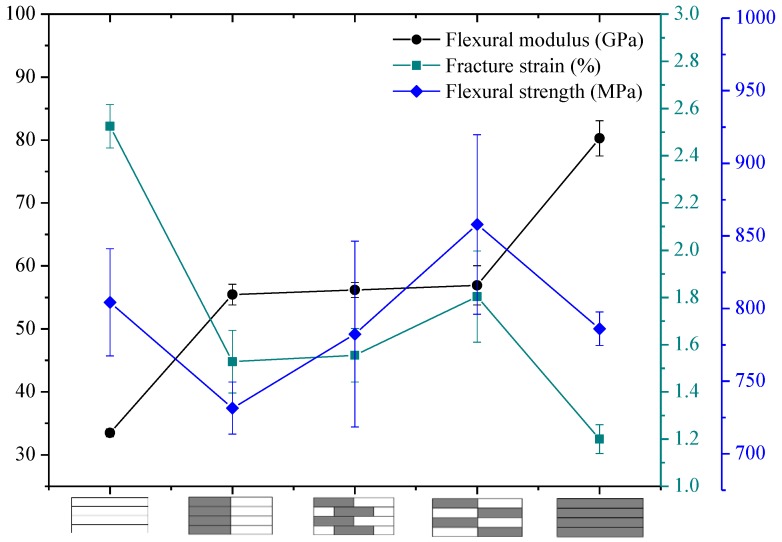
Flexural modulus, fracture strain, and strength of intralayer hybrid composite with various stacking sequences at C/G = 5 mm:5 mm.

**Figure 17:**
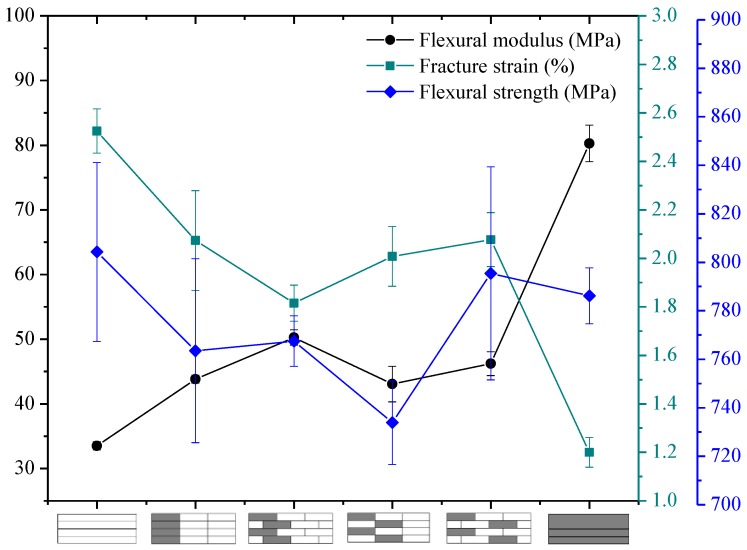
Flexural modulus, fracture strain, and strength of intralayer hybrid composite with various stacking sequences at C/G = 5 mm:10 mm.

**Figure 18:**
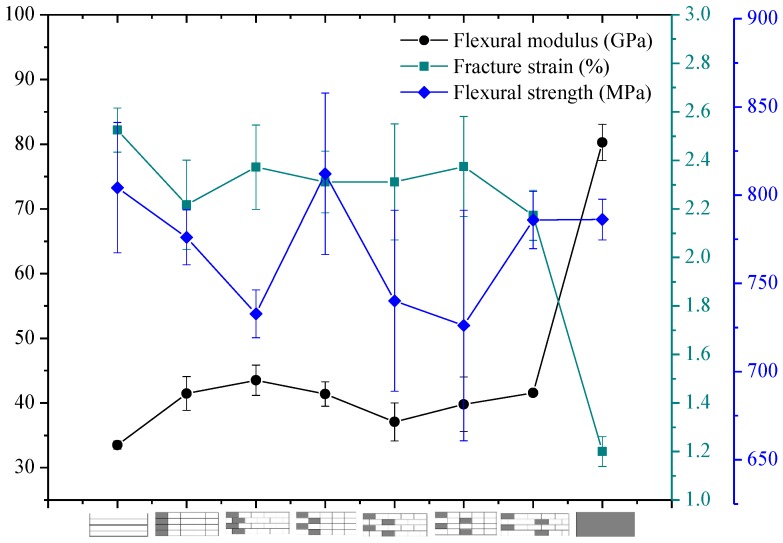
Flexural modulus, fracture strain, and strength of intralayer hybrid composite with various stacking sequences at C/G = 5 mm:20 mm.

The flexural properties of intralayer hybrid composites with various hybrid ratios and structures were tested in this section, and the results are presented in Figure 16, Figure 17 and Figure 18.

Figure 16 exhibits the bending properties of intralayer hybrid composites with C/G = 5 mm:5 mm. As shown here, the flexural modulus of hybrid composites is between that of carbon fiber and glass fiber composites and maintains a relatively stable level. The bending strength and fracture strain increases slightly with the increase of the C/G hybrid dispersion degree.

As observed in Figure 16, Figure 17 and Figure 18, the flexural fracture strain, strength, and modulus of intralayer hybrid composites with the same C/G mixed ratio show a weak fluctuation and remain at the same level with the increase of the C/G mixed dispersion. 

**Figure 19:**
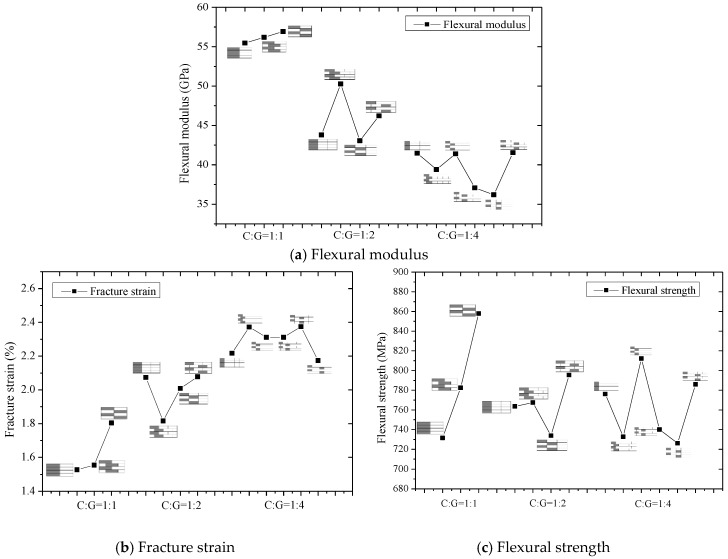
Flexural properties comparisons for various stacking sequences under three kinds of C/G ratios.

Figure 19 compares the flexural modulus, strength, and fracture strain of intralayer hybrid composites. The flexural strain of intralayer hybrid composites increases as the carbon fiber content decreases overall, whereas the flexural modulus shows an opposite trend. Under the same hybrid ratio, the flexural modulus of hybrid composites with various hybrid structures changes slightly and keeps the same level. Moreover, the flexural strength is independent of the carbon fiber fraction except the C:G = 1:1. 

The mechanical properties of hybrid composites exhibit different hybrid effects. While some hybrid forms enhance their performances, some may weaken their performances. Therefore, the rule of mixture (ROM) is introduced to evaluate the hybrid effect. 

The ROM is a method to calculate the mechanical property of hybrid composites according to the content of two materials [21]:

(6) $$\varepsilon_{\mathrm{FROM}}=\varepsilon_{\mathrm{FC}}V_{C}+\varepsilon_{\mathrm{FG}}V_{G}$$

(7) $$E_{\mathrm{ROM}}=E_{C}V_{C}+E_{G}V_{G}$$

(8) $$\sigma_{\mathrm{ROM}}=\sigma_{C}V_{C}+\sigma_{G}V_{G}$$

where $\varepsilon_{\mathrm{FROM}}$, $\varepsilon_{\mathrm{FC}}$, $\varepsilon_{\mathrm{FG}}$ denote the flexural fracture strain of hybrid composite, carbon fiber composite, and glass fiber composite, respectively. $E_{\mathrm{ROM}}$, $E_{C}$, $E_{G}$ refer to the flexural modulus of hybrid composite, carbon fiber composite, and glass fiber composite (GPa), respectively. $\sigma_{\mathrm{ROM}}$, $\sigma_{C}$, $\sigma_{G}$ represent the flexural strength of hybrid composite, carbon fiber composite, and glass fiber composite (MPa), respectively. $V_{C}$, $V_{G}$ are the volume content of carbon fiber and glass fiber composites.

**Figure 20:**
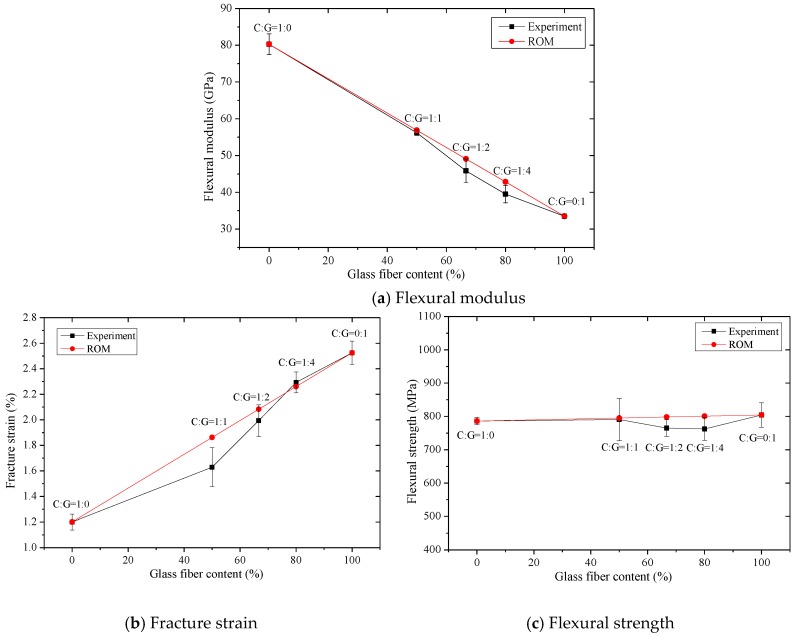
Flexural properties obtained via the experimental and the ROM methods for intralayer hybrid composites.

The flexural fracture strain, modulus, and strength of intralayer hybrid composites, presented in Figure 20, are basically consistent with the calculated values via the ROM. As the glass fiber content increases, the flexural modulus decreases linearly, the bending fracture strain increases linearly, and the flexural strength appears unchanged. It is indicated that the ROM is a reasonable method for obtaining the flexural properties for intralayer hybrid composites. Compared with the experimental values and ROM values, it was found that the bending properties of the intralayer hybrid composites exhibit strong or negative hybrid effects, in which the flexural modulus and strength exhibit a weak negative hybrid effect, but the fracture strain shows a strong negative hybrid effect, indicating that the intralayer hybrid structures reduce the bending performances of hybrid composites.

### 3.3. Comparison of Flexural Properties between Interlayer and Intralayer Hybrid Composites 

**Figure 21:**
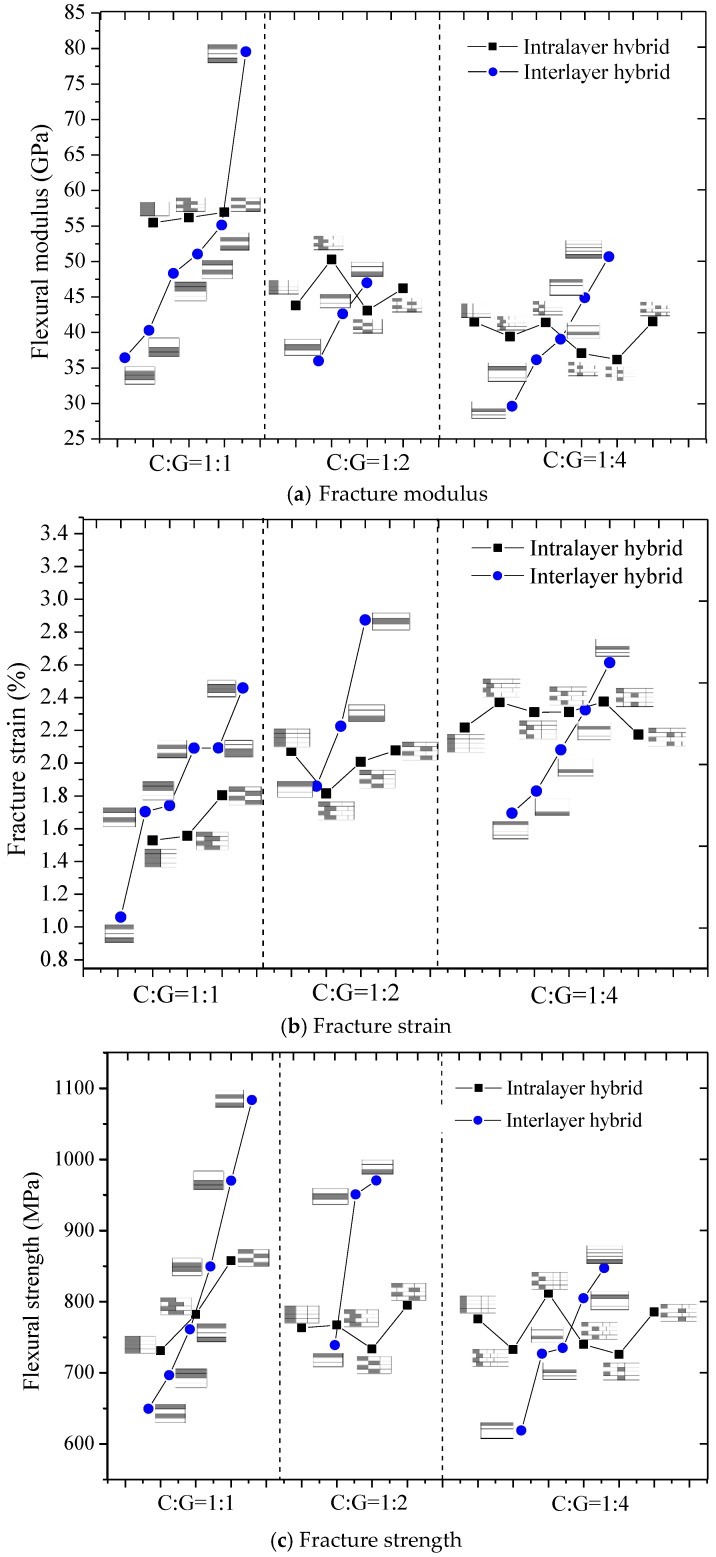
Flexural properties of interlayer and intralayer hybrid composites.

Flexural properties between the interlayer and intralayer hybrid composites were compared as seen in Figure 21. From the comparison of the flexural modulus in Figure 21a, as the glass fiber content increases, the modulus of interlayer and intralayer hybrid composites gradually decreases, but the effect on the modulus of the interlayer composites caused by the layer structure exceeds the effect of hybrid ratio, which indicates the interlayer structure exhibits a stronger designability. For intralayer hybrid composites, the effect of layup structure on the flexural modulus is unclear. In addition, as the hybrid composites contain more carbon fiber, like the C:G = 1:1, the interlayer hybrid structures exhibit a higher average flexural modulus than the interlayer structures except the [C/G/G/C]. 

From the bending fracture strain of interlayer and intralayer hybrid composites shown in Figure 21b, it was found that as the glass fiber content increases, the bending fracture strain of hybrid composites shows an opposite trend compared with the flexural modulus. The fracture strain of interlayer hybrid composites is mainly affected by the layer structure, with regards to the intralayer hybrid composites, the hybrid ratio is the main reason to influence the flexural strain, while C:G = 1:1, 1:2, a high dispersion degree also improves the flexural strain. 

As seen from Figure 21c, it could be found that the bending strength is independent of the hybrid ratio for both interlayer and intralayer hybrid composites, while the layer structure has a significant influence on the interlayer structures which indicates the interlayer composites exhibit a better designability than the intralayer composites.

Therefore, the decisive factor on the bending properties for interlayer hybrid composites is this: layer structure > C/G mixed ratio. For intralayer hybrid composites, the C/G mixed ratio has a decisive impact on the flexural fracture strain and modulus; however, the flexural properties are affected weakly by the layer structure. Moreover, better flexural properties for interlayer hybrid composites can be achieved by the layer structure optimization with less carbon fiber.

## 4. Conclusions

In this paper, flexural properties of interlayer and intralayer hybrid composites with various hybrid structures and mixed ratios were studied. First, the theoretical stress distribution of interlayer hybrid structures was discussed, and apparent flexural properties were adopted to compare hybrid composites with various structures and hybrid ratios. From the experimental results, the decisive factors for flexural properties of interlayer hybrid composites are determined by the compressive property of upper layers and the tensile property of lower layers; therefore, the layer structure plays an important role in the flexural property of interlayer hybrid composites, while the effect of the hybrid ratio on the flexural properties is small. With the carbon fiber distributed in the exterior layer, the flexural modulus of interlayer hybrid composites of the 1:1 C/G mixed ratio is maximum, and an excellent flexural strength can be achieved with the carbon fiber located in the bottom layer. Whereas the interlayer hybrid structure contains only one carbon layer such as the C:G = 1:2, 1:3, 1:4, the flexural modulus and strength with the carbon fiber distributed at the bottom layer are higher. 

With regards to intralayer hybrid composites, the flexural fracture strain, strength, and modulus of intralayer hybrid composites with the same C/G mixed ratio show a weak fluctuation and remains at the same level with the increase of the C/G mixed dispersion; as the carbon fiber content increases, the flexural fracture strain decreases, the flexural modulus increases, and the flexural fracture strength basically maintains at a steady level. In addition, the experimental results on the intralayer composites comply well with the theoretical values calculated via the ROM, and the flexural properties all exhibiting the negative hybrid effects indicate that the intralayer hybrid structures weaken the bending performance of hybrid composites. 

In general, the better designability of the interlayer hybrid composites as compared to the intralayer hybrid structures makes it possible to achieve excellent flexure performances with less carbon fiber through the optimization of the mixed ratio and the hybrid structure. Thus, the flexural properties of hybrid composites can reach a higher level.

## Figures and Tables

**Table 1:** Specifications for hybrid fabric.

Fabric type	Areal density (g/m^2^)		Ratio of C/G
	Carbon fiber	Glass fiber	
Carbon	728.3	0	1:0
Glass	0	944.9	0:1
C-G	364.2	472.4	1:1
C-G-G	242.8	629.9	1:2
C-G-G-G-G	145.7	755.9	1:4

**Table 2:** Constituent materials and selected properties.

Material	Tensile strength (MPa)	Tensile modulus (GPa)
CPIC ECT469L-2400 Glass Fiber	2366	78.7
TORAY T620SC-24K-50C Carbon Fiber	4175	234
SWANCOR 2511-1A/BS Epoxy Resin	73.5	3.1

**Table 3:** Stacking configurations of interlayer hybrid structures.

C/G Hybrid ratios	Stacking sequences					
C:G=1:1	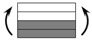	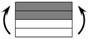	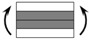	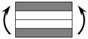	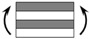	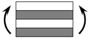
	[G/G/C/C]	[C/C/G/G]	[G/C/C/G]	[C/G/G/C]	[C/G/C/G]	[G/C/G/C]
C:G=1:2	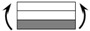	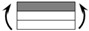	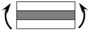			
	[G/G/C]	[C/G/G]	[G/C/G]			
C:G=1:3	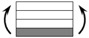	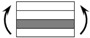	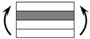	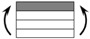		
	[G/G/G/C]	[G/G/C/G]	[G/C/G/G]	[C/G/G/G]		
C:G=1:4	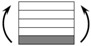	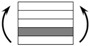	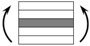	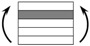	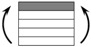	
	[G/G/G/G/C]	[G/G/G/C/G]	[G/G/C/G/G]	[G/C/G/G/G]	[C/G/G/G/G]	

Note: the arrow in Table 3 and Table 4 indicates the flexural loading.

**Table 4:** Stacking configurations of intralayer hybrid structures.

C/G Hybrid ratios	Ply sequences					
C-G C:G=1:1	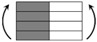	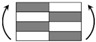		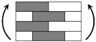		
	[C-G]-0	[C-G]-1		[C-G]-0.5		
C-G-G C:G=1:2	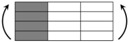	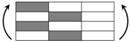		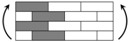		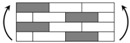
	[C-G-G]-0	[C-G-G]-1		[C-G-G]-0.5		[C-G-G]-1.5
C-G-G-G-G C:G=1:4	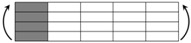		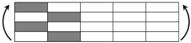		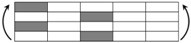	
	[C-G-G-G-G]-0		[C-G-G-G-G]-1		[C-G-G-G-G]-2	
	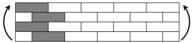		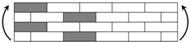		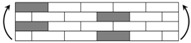	
	[C-G-G-G-G]-0.5		[C-G-G-G-G]-1.5		[C-G-G-G-G]-2.5	

**Table 5:** Size parameters of composites.

Laminate structures	C/G Hybrid ratios	Layers	Laminate thickness/mm	Width/mm	Span/mm
Pure carbon fabric	1:0	4	3.2	13	64
Pure glass fabric	0:1	4	3.2	13	64
Interlayer laminate	1:1	4	3.2	13	64
	1:2	3	2.4	13	48
	1:3	4	3.2	13	64
	1:4	5	4	13	80
Intralayer laminate	1:1	4	3.2	20	64
	1:2	4	3.2	15	64
	1:4	4	3.2	25	64
